# Economic evaluation alongside a single RCT of an integrative psychotherapeutic nursing home programme

**DOI:** 10.1186/1472-6963-13-370

**Published:** 2013-09-30

**Authors:** Leona Hakkaart-van Roijen, Ton JEM Bakker, Maiwenn Al, Jacqueline van der Lee, Hugo J Duivenvoorden, Miel W Ribbe, Robbert Huijsman

**Affiliations:** 1 Institute for Medical Technology Assessment (iMTA), Erasmus MC – University Medical Center Rotterdam, Rotterdam, the Netherlands; 2 Psychiatric-skilled nursing home 'DrieMaasStede’, Argos Zorggroep, Voorberghlaan 35, P.O. Box 4023 3102, GA Schiedam, The Netherlands; 3 VU Department of Nursing Home Medicine, EMGO Institute for Health and Care Research VU University Amsterdam/VU University Medical Center, Amsterdam, The Netherlands

**Keywords:** Economic evaluation, Nursing home, Cognitive impairment, Neuropsychiatric symptoms, Psychiatry, BPSD, Psychotherapy

## Abstract

**Background:**

There is an 80% prevalence of two or more psychiatric symptoms in psychogeriatric patients. Multiple psychiatric symptoms (MPS) have many negative effects on quality of life of the patient as well as on caregiver burden and competence. Irrespective of the effectiveness of an intervention programme, it is important to take into account its economic aspects.

**Methods:**

The economic evaluation was performed alongside a single open RCT and conducted between 2001 and 2006. The patients who met the selection criteria were asked to participate in the RCT. After the patient or his caregiver signed a written informed consent form, he was then randomly assigned to either IRR or UC.

The costs and effects of IRR were compared to those of UC. We assessed the cost-utility of IRR as well as the cost-effectiveness of both conditions. Primary outcome variable: severity of MPS (NPI) of patients; secondary outcome variables: general caregiver burden (CB) and caregiver competence (CCL), quality of life (EQ5D) of the patient, and total medical costs per patient (TiC-P). Cost-utility was evaluated on the basis of differences in total medical costs). Cost-effectiveness was evaluated by comparing differences of total medical costs and effects on NPI, CB and CCL (Incremental Cost-Effectiveness Ratio: ICER). CEAC-analyses were performed for QALY and NPI-severity. All significant testing was fixed at p<0.05 (two-tailed). The data were analyzed according to the intention-to-treat (ITT)-principle. A complete cases approach (CC) was used.

**Results:**

IRR turned out to be non-significantly, 10.5% more expensive than UC (€ 36 per day). The number of QALYs was 0.01 higher (non-significant) in IRR, resulting in € 276,290 per QALY. According to the ICER-method, IRR was significantly more cost-effective on NPI-sum-severity of the patient (up to 34%), CB and CCL (up to 50%), with ICERs varying from € 130 to € 540 per additional point of improvement.

**Conclusions:**

No significant differences were found on QALYs. In IRR patients improved significantly more on severity of MPS, and caregivers on general burden and competence, with incremental costs varying from € 130 to € 540 per additional point of improvement. The surplus costs of IRR are considered acceptable, taking into account the high societal costs of suffering from MPS of psychogeriatric patients and the high burden of caregivers. The large discrepancy in economic evaluation between QALYs (based on EQ5D) and ICERs (based on clinically relevant outcomes) demands further research on the validity of EQ5D in psychogeriatric cost-utility studies. (*Trial registration nr.: ISRCTN 38916563; December 2004*).

## Background

In psychogeriatric patients who suffer from cognitive impairment or dementia, there is an 80% prevalence of two or more psychiatric symptoms, e.g. depression, anxiety, paranoia, aggression [1,2]. Multiple psychiatric symptoms (MPS) have many related negative effects on the quality of life of the patients as well as on caregiver burden and competence [1,3,4]. Psychotropic drugs, e.g. (a) typical antipsychotics, are widely used to treat the MPS in usual nursing home care, despite their limited effects and potentially harmful side-effects [5,6]. Reports in the literature indicate that psychotherapeutic treatment may be effective for individual psychiatric symptoms [7]. However, psychotherapeutic interventions that focus on MPS are complex, due to the multiple nature of MPS in combination with cognitive disorders, somatic co-morbidity, and social problems. Furthermore, our literature search did not reveal any large-scale, comprehensive randomized controlled (RCT) studies on integrative psychotherapeutic programmes in nursing homes [8,9]. For these reasons, we developed a unique integrative psychotherapeutic nursing home programme: integrative reactivation and rehabilitation (IRR) [10]. The IRR-programme was aimed at psychogeriatric patients who were at high risk for admission to a nursing home. Limits on health-care resources mandate that resource-allocation decisions are guided by considerations of costs in relation to expected benefits. In cost-effectiveness analysis, the ratio of net health-care costs to net health benefits provides an index by which priorities may be set. Quality-of-life concerns are commonly incorporated in the calculation of health benefits as adjustments to life expectancy [11]. The incremental cost-effectiveness ratio (ICER) is the ratio of the estimated difference between the costs of two interventions and the estimated difference between the outcomes of these two interventions. Cost-effectiveness studies are mainly used to facilitate informed decision making about interventions that are both more costly and more effective than their comparator. The performed RCT was designed to test the (cost-effectiveness) of IRR on MPS of psychogeriatric patients as well as on burden and competence of the caregiver. The primary analyses concerned the mean differences between IRR and the usual multidisciplinary nursing home care (UC) on continuous data of the outcome variables. The results of this analysis are published elsewhere [12]. IRR had a significant and moderate to large surplus effect (up to 34%) on reducing the MPS of psychogeriatric patients. In fact, at six-month follow-up there was a total reduction up to 46% in number and 61% in severity. Furthermore, at the end of the treatment IRR had a large surplus effect (up to 36%) on reducing caregiver burden. During the follow-up the surplus effect even increased up to 50%, while UC showed almost no effect. Irrespective of their effectiveness, the economic aspect of treatment programmes, i.e. an economic evaluation from a societal perspective, is also important [13]. The costs and effects of IRR were compared to those of UC. We assessed the cost-utility of IRR as well as the cost-effectiveness of both conditions on three outcome variables.

## Methods

### Patients

In the urban region of Nieuwe Waterweg Noord (NWN), near Rotterdam in the Netherlands, the patients were referred from an (ambulant) mental health service (5.4%), a general hospital (13.8%), a memory clinic (6%) and by general practitioners or primary healthcare services (75.1%). Before inclusion in the randomized controlled trial, all patients underwent a comprehensive assessment. The inclusion criteria were: 1) Diagnostic and Statistical Manual (DSM IV) classification of dementia, amnestic disorders or other cognitive disorders; 2) age: ≥65 years; 3) psychiatric symptoms: Neuropsychiatric Inventory (NPI) 3 or more symptoms; 4) cognitive functioning: Mini Mental Status Examination (MMSE) ≥18 and ≤27 as well as Barthel Index (BI) ≥5 and ≤19, and 5) informed consent. The exclusion criteria were: 1) delirium; 2) life-threatening somatic co-morbidity; 3) active compulsory admission regime (according to psychiatric legislation), and 4) insufficient command of the Dutch language.

### Design

The economic evaluation was performed alongside an open Randomised Controlled Trial (RCT), conducted between 2001 and 2006. Included patients were randomly and blindly assigned to either IRR or UC, using a randomisation algorithm. In the first half of the study the assignment ratio was 1 (IRR):2 (UC). However, due to the limited number of eligible patients, time restrictions and financial limitations, this was reversed to 2 (IRR):1 (UC) in the second half of the study. We ultimately included 168 patients (81 IRR and 87 UC). 'Multiple psychiatric symptoms of the patient’ was the primary outcome variable, and 'Burden’ and 'Competence’ of the caregiver were secondary outcome variables. For the economic evaluation we collected the data of direct medical costs of the patient. The outcome variables were assessed at T1 (within two weeks after intake) and at T3 (follow-up; six months after the conclusion of the three-month intervention). Measurements of the costs were conducted every 8 weeks over the previous four weeks. The final measurement took place at 40 weeks. Data were collected by trained assessors who were not members of the Intervention team. Assessors were not blind to the intervention. This study was approved by the Medical Ethics Review Committee (METC) of the Erasmus University Medical Centre.

### Intervention

**Figure 1:**
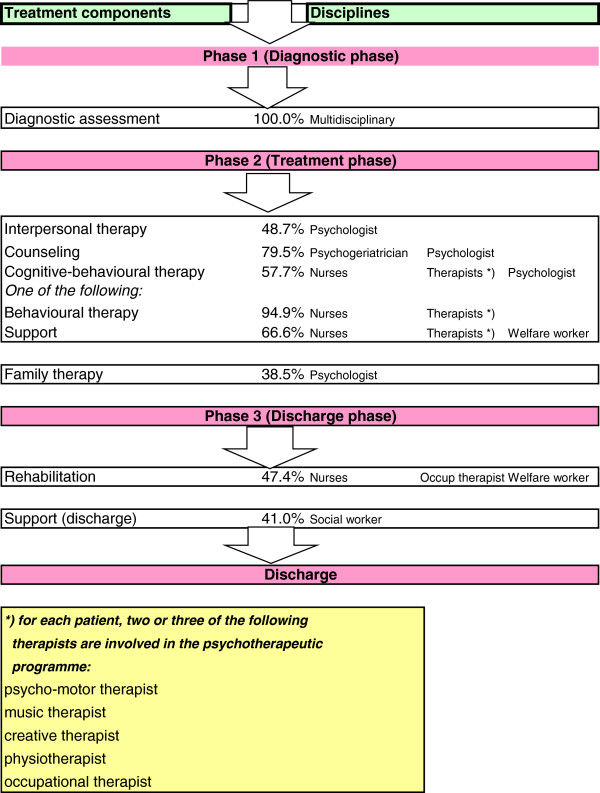
Flowchart of IRR treatment programme, distinguished by three phases.

The duration of IRR programme was 13 weeks, with clinical admission to a separate 15-bed specialized unit in a psychiatric-skilled nursing home. IRR is meant as a short-stay reactivation and rehabilitation programme in addition to the usual multidisciplinary nursing home care, including psychotropic drug treatment. IRR consisted of both integrative psychotherapeutic interventions to treat multiple psychiatric symptoms of the patient, and family therapy for the caregiver. Furthermore, both cognitive and somatic functioning were optimized. The patients followed the IRR program in three phases: 1. diagnostics, 2. treatment, and 3. discharge (Figure 1). The multidisciplinary IRR team consisted of a nursing team, a psychogeriatrician, a clinical psychologist, a social worker, a music therapist, a psychomotor therapist and a creative therapist, a physiotherapist, an occupational therapist, a speech therapist, a dietician and a welfare worker [12]. In the diagnostic phase (phase 1) a personal package of interventions was put together for each patient and caregiver, based on selected specific functional problems on six dimensions: 'Emotion’ (e.g. depression, anxiety, aggression), 'Personality’ (e.g. characteristics of narcissism, borderline personality disorder, dependency), 'Life events’ (e.g. traumatic experiences such as war, incest, death of a spouse/child), 'Social functioning’ (e.g. relationship problems with spouse/children, loss of pleasant social activities), 'Cognitive functioning’ (e.g. problems with memory, self-care), and 'Somatic functional disorders’ (e.g. impaired mobility, falls, polypharmacy, nutritional deficiency and intercurrent diseases) [10]. In the treatment phase (phase 2) and the discharge phase (phase 3), the following psychotherapeutic interventions - which were based on a problem-solving theoretical framework and recorded in guidelines for each discipline - were applied: 1) diagnostic assessment, 2) counselling, 3) life-review, 4) interpersonal therapy, 5) cognitive-behavioural therapy, 6) behavioural therapy, 7) support in accepting behaviour and minimizing negative effects, 8) regression approach, temporarily accepting regression behaviour, 9) rehabilitation, 10) support from social worker on discharge, 11) psycho-education, and 12) family therapy [10,12]. The percentages in Figure 1 correspond to the percentages of patients who participated in the specific intervention during the IRR phases. A patient could participate in different interventions prescribed for the specific functional problems.

Interventions were provided mainly in a group setting, but also individually when necessary. On average patients received treatment for five functional psychiatric problems, provided by four disciplines; sometimes a discipline used more than one type of intervention. The availability of a wide range of interventions made it possible to provide a highly individualized package of interventions. After multidisciplinary consultation, the psychogeriatrician - a nursing home physician with experience in psychiatric treatment - prescribed the interventions for each functional problem. Staff members were trained to systematically conduct the IRR programme. Monitoring took place weekly during the course of the personal intervention plan and was guided by the method of standardized goal attainment scaling (GAS: score range 1 to 7; 7 = independent (no help needed) [14]. Each discipline provided a written GAS score, based on functional progress during the therapy sessions. These scores were discussed in the multidisciplinary meeting, without the patients/caregivers, and a consensus GAS score was determined.

UC consisted of a relatively high level of nursing home care. This care is provided by a multidisciplinary team. In this study, UC was provided in the following settings: at home (n=22; 25.3%), at home with mental healthcare (out-reaching) or psychogeriatric day care/treatment (n=14; 15.7%), in an assisted living residence (n=6; 7.2%), and in a nursing home (n=45; 51.8%). The multidisciplinary UC staff consisted of a nursing home physician or social geriatric physician, a psychologist, a paramedical team (physiotherapist, occupational therapist, speech therapist, welfare worker) and a nursing team (Registered Nurses, Certified Nurse Assistants and/or Nurse Assistants). UC was provided by different types of core multidisciplinary teams, each working from a different, mostly emotion-oriented, theoretical framework.

### Assessments

We used two sets of instruments, one for the patient and caregiver, and one for the economic aspects. Multiple psychiatric Symptoms (MPS) of the patient was assessed by the Neuropsychiatric Inventory (NPI 12 item version: *'NPI-sum-severity’* : 0 to 144; 0 = no severity symptoms at all), administered to the caregiver [15]. We chose the caregiver NPI, because the caregivers formed a stationary group from the moment of inclusion in the study until the end of the treatment and the six-month follow-up. Furthermore, they generally provide a better reflection of the actual needs, thoughts and feelings of dementia patients than the relatively discontinuous nursing teams can provide [16]. General burden of the caregiver was assessed using the Caregiver Burden (CB: 0 to 100; 0=optimal) [17], and competence with the Caregiver Competence List (CCL: 28 to 112; 112=optimal) [4,18]. MMSE was used to measure patient memory (0 to 30; 30=normal) [19], and self-care was assessed using the Barthel Index (BI) (0 to 20; 20=normal) [20]. To assess the risk for being placed in a nursing home we used the Global Deterioration Scale (GDS: 1–7; 1 is normal) [21]. Somatic co-morbidity was assessed using the International Classification of Diseases (ICD-10). The DSM IV disorders (axes I and II) were classified by a research psychiatrist. The following demographic data were collected: gender, age, marital status, family relation, residence, education level, income level and job employment.

For the economic evaluation, i.e. cost utility analysis, we assessed quality of life with EuroQol Health questionnaire *(*EQ5D) (-0.59 to 1.0; 1.0=optimal), administered to the patient [22]. The EQ5D is a validated tool for measuring general health–related quality of life. EQ5D consists of five items (mobility, self-care, usual activities, pain/discomfort and anxiety/depression), each having the rating options 'no problems’, 'some problems’ and 'extreme problems’. The health descriptions can be linked directly to empirical valuations of the general public, which allows utilities to be computed [23].

Costs were estimated by multiplying the use of health care by their corresponding unit prices. The number of days admitted to the nursing home was collected directly from the participating centres. The questionnaire Trimbos iMTA for Costs associated with Psychiatric illness (TiC-P), which asks about the number of health care contacts over the previous four weeks, was applied to collect data on all other use of health care from the patients. We used TiC-P to collect data on direct medical costs [24]. Unit prices of the interventions for the year 2004 were estimated based on information provided by the financial department of Argos Zorggroep. Therefore, data on the direct (e.g. medical staff, nursing staff) and indirect costs (e.g. overhead, housing) of 2004 was used to calculate the unit costs per day for IRR and UC, respectively. All other health care utilisation was valued using their corresponding unit prices based on the Dutch manual for costing studies in Economic Evaluations [25]. Unit prices of health care services for 2004 were adjusted to 2005 prices using the consumer price index (http://www.cbs.nl). So, all relevant health care costs were included.

The patient mean utility scores were estimated by applying the area-under-the curve method (AUC) [25]. The data scores of patients who died were valued zero if the patient died in the first 4 weeks of a measurement period or in any of the consecutive measurement periods. If a patient died in the last 4 weeks of a measurement period we valued the data scores as missing, or the available scores of the measurement period in which the person died, and zero in the consecutive periods.

### Economic evaluation

The cost-utility was evaluated by relating the difference in total direct medical costs per patient to the difference in terms of Quality Adjusted Life Years gained (QALY), which yielded a cost per QALY estimate. In addition, to estimate the cost effectiveness of IRR versus UC, we determined the Incremental Cost-Effectiveness Ratio (ICER) by comparing the two conditions on mean differences in total medical costs divided by mean differences in effects on NPI, CB and CCL, respectively [26]. The estimated participation interval of dropouts (time in days participating in the study) was determined using a Cox-regression analysis. All significance testing was fixed at P<0.05 (two-tailed). The data were analyzed according to the intention to treat (ITT)-principle. The statistical analyses were performed with the software programmes SPSS, version 21, and SAS, version 9.2.

## Results

### Characteristics of the study sample

**Figure 2:**
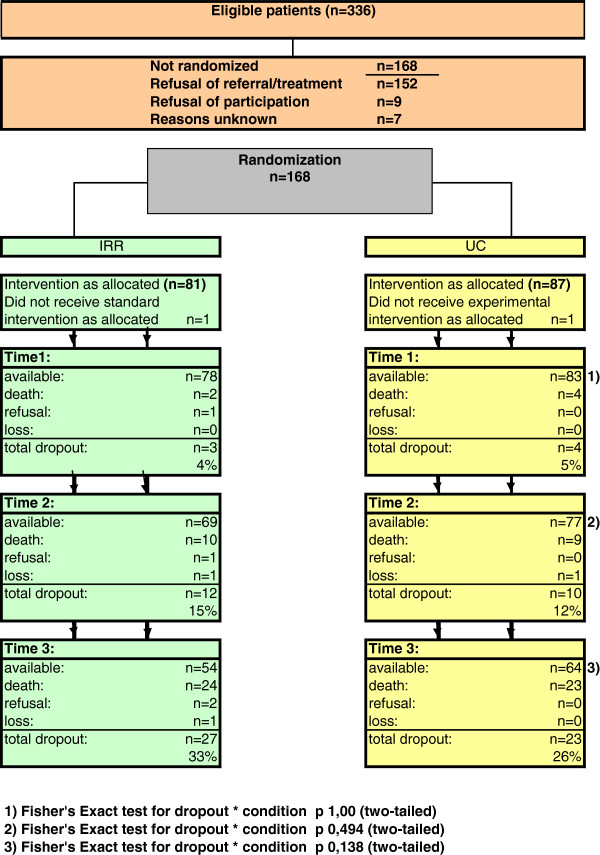
Flowchart study sample, distinguished by treatment condition.

Of the 336 eligible individuals, 168 (50%) consented to participate in the RCT (Figure 2). The non-participants did not differ significantly from the participants on the inclusion criteria. The difference between the two study groups in number of dropouts was insignificant. Moreover, the dropouts did not differ significantly with regard to any baseline assessment or on length of time participating in the programme (Cox regression analysis: HR 1.21; P<0.54).

The IRR group and UC group differed significantly only on somatic co-morbidity (Table 1). All statistical analyses were adjusted for somatic co-morbidity. A mean GDS-score of 4.2 (SD 0.8) suggested that the study sample consisted of psychogeriatric patients with mild cognitive impairment or dementia who were at high risk for admission to a nursing home [27]. The mean sum-severity of NPI-symptoms of the patient (IRR 35.90 and UC 29.68) was relatively high (Table 2). Kaplan-Meier analysis showed that the length of stay in a nursing home after the end of treatment did not differ significantly between the two groups: IRR mean (147.04 days, 95% CI: 123.77 to 170.31), and UC mean (151.82 days; 95% CI: 130.11 to 173.37; p<0.62) (Table 3).

**Table 1:** General details of participants, distinguished by intervention

	**IRR**	**UC**		
	**n=81**	**n=87**	**p-value**	
***Patient characteristics***				
**Gender (females)**	66.7%	62.1%	0.63	^1)^
**Age (in years), mean (SD)**	79.8 (6.1)	81.5 (7.1)	0.10	^2)^
**Marital status: alone**	77.8%	80.5%	0.71	^1)^
**Educational level: low**	67.5%	68.7%	0.90	^1)^
**Domicile: at home**	76.5%	66.7%	0.17	^1)^
**Primary caregiver: spouse**	17.3%	13.8%	0.33	^1)^
**DSM-IV dementia, (axis-I), count (%):**				
**Dementia of the Alzheimer’s type**	18.5%	17.2%	0.84	^1)^
**Vascular dementia**	23.5%	25.3%	0.86	
**Dementia due to other conditions**	16.0%	19.5%	0.69	
**Amnestic/cognitive disorders**	32.1%	31.0%	1.00	
**Other**	6.2%	2.3%	0.26	
**DSM-IV personality disorders (axis-II), count (%)**	16.0%	9.2%	0.24	
**MMSE**	20.0(4.5)	20.9(3.8)	0.42	^2)^
**Barthel index**	15.4(3.8)	14.7(3.6)	0.25	
**GDS-deterioration, mean (SD)**	4.2 (0.7)	4.3 (0.9)	0.62	
**Somatic co-morbidity (ICD-10), mean (SD)**	5.6 (2.6)	4.5 (2.4)	0.01	^2)^
**Nursing home days (LOS), mean (SD)**	147.0 (107.0)	151.8 (103.9)	0.62	^2)^
***Caregiver characteristics***				
**Gender (females)**	70.5%	61.7%	0.32	^1)^
**Age (in years), mean (SD)**	58.6 (11.9)	58.9 (12.0)	0.86	^2)^
**Marital status: living together**	91.4%	94.8%	0.52	^1)^
**Educational level: low**	4.3%	2.6%	0.39	^1)^

^1)^Fisher’s exact test (two tailed).

^2)^*t*-Test (two tailed).

**Table 2:** Level of outcome across time (T1 - T3) distinguished by intervention

			**T1 (baseline measurement)**									**T3 (six months follow-up)**			
			**IRR**			**UC**						**IRR**		**UC**	
	**Range**	**High score**	**n**	**Mean**	**SD**	**n**	**Mean**	**SD**	**Mean**	**95% CI**		**n**	**Mean**	**n**	**Mean**
		= (+/-) *)							**diff.**						
***Psychiatric function disorders patient***															
***(by caregiver)***															
**NPI-sum-severity**	0 to 144	(-)	72	35.90	21.84	76	29.68	20.12	-6.22	-13.05	0.62	49	15.84	51	18.61
***Caregiver burden***															
**Caregiver burden (CB)**	0 to 100	(-)	72	52.47	25.65	77	46.69	27.66	-5.79	-14.42	2.85	42	28.81	50	44.90
**Competence (CCL)**	28 to 112	(+)	72	84.62	14.24	77	86.58	14.46	1.96	-2.69	6.6	49	96.35	50	91.78
***Quality of life patient***															
**EQ5D**	-0.59 to 1.00	(+)	77	0.54	0.34	80	0.58	0.29	0.04	-0.06	0.14	45	0.62	62	0.55

*) + = high score is beneficial, - = high score is unfavourable.

**Table 3:** Mean direct medical costs (€) and mean QALY after 40 weeks for IRR and care as usual

**Complete case analysis (CC)**									
							**Mean**		
	**Mean IRR**^**1)**^	**95%**	**CI**	**Mean UC**^**2)**^	**95%**	**CI**	**UC-IRR**	**95%**	**CI**
***Week 40***									
**Home care**	21.05	0.00	65.01	2895.01	1214.53	4825.43	2873.96	1182.33	4807.01
**Day care**	0.00	0.00	0.00	2279.18	730.57	4328.72	2279.18	730.57	4328.72
**Hospital**	964.74	259.62	1873.95	420.34	0.00	913.12	-544.40	-1768.63	422.81
**Nursing home (incl. IRR)**	27675.61	22546.21	32545.24	20123.51	14258.25	26691.78	-7552.10	-15927.53	-8971.61
**Assisted living residence**	4902.63	2711.53	7396.54	4033.14	1643.42	6457.91	-869.49	-4335.40	2538.42
**Other care**	149.16	74.48	243.83	757.05	259.28	1338.68	607.89	91.75	1207.87
**QALY**	0.43	0.36	0.51	0.42	0.36	0.48	0.013 ^3^	-0.09	0.11
**Direct medical costs**	33713.00	27961.18	39220.66	30508.00	25696.53	35270.19	-3204.95	-10326.22	4812.52

^1)^Integrative reactivation and rehabilitation.

^2)^Usual care.

^3)^Costs per Qaly € 276,289.70.

### Economic evaluation

At baseline, TiC-P and EQ5D data were available for 96% (n=161), and at 40 weeks follow-up for 38% (n=63) of the participants. Table 3 shows that the mean nursing home costs per patient (including the costs of IRR) were non-significantly higher in IRR than in UC. However, the costs of home care and day care were significantly lower in IRR. The mean costs in IRR were non-significantly (10.5%) higher (€ 3,205; 95% CI: 374.20 to 10153.20) than those in UC. This implies € 36 extra per IRR-treatment day (in total: average 90 days treatment duration). Moreover, the number of QALYs of the patients was non-significantly (0.01) higher in IRR (95% CI:-0.09 to 0.11), which resulted in a mean of € 275,000 per QALY.

Table 4 presents the cost-effectiveness of IRR in ICERs. IRR was significantly more effective on the primary outcome variable NPI-sum-severity of the patient as well as on the secondary outcome variables caregiver burden and caregiver competence. The effects of IRR were about twice as large as of those of UC. For NPI-sum-severity, the ICER equalled to about € 320.--, implying that the cost of one surplus point improvement in IRR was € 320 (mean difference IRR-UC=10 points). The least expensive was improvement on general burden of the caregiver, ICER € 130 (mean difference 25 points). The ICER of competence of the caregiver was € 540 (mean difference 6 points).

**Table 4:** Cost-effectiveness after 40 weeks; distinguished by Intervention; ICER-approach

	**High score**	**Costs**		**Effects**		**Costs**		**Effects**		**ICER**
	=(+/-) *)	**Costs IRR**	**Costs UC**	**IRR**	**UC**	**IRR-UC**	**p-value**	**IRR-UC**	**p-value**	**Costs/effects**
**WEEK 40**										
**NPI-sum-severity**	(-)	33713.19	30508.23	21.78	11.86	3204.95	0.37	9.92	0.04	323.08
**Caregiver burden (CB)**	(-)	33713.19	30508.23	24.76	0.00	3204.95	0.37	24.76	0.00	129.44
**Caregiver competence (CCL)**	(+)	33713.19	30508.23	10.35	4.42	3204.95	0.37	5.93	0.01	540.46

*) + = high score is beneficial, - = high score is unfavourable.

The cost-effectiveness acceptability curve (CEAC) illustrates the uncertainty surrounding the estimate of cost- effectiveness [28-32]. CEACs are intended to represent the uncertainty concerning the cost-effectiveness of a health-care intervention in the context of decision making involving two interventions as an alternative to confidence intervals around ICERs [28]. Nowadays, use of CEACs has become widespread amongst others in the field of medicine [33-37].

The CEAC is based on the joint density of incremental costs (ΔC) and incremental effects (ΔE) for the intervention of interest (IRR), and represents the proportion of the density where the intervention is cost-effective for a range of values of λ. In our study, the CEAC is estimated by parametric bootstrapping of the distribution [28,29,31]. The CEAC is determined as the proportion of the (ΔC/ ΔE) points where the intervention is considered cost-effective [29].

**Figure 3:**
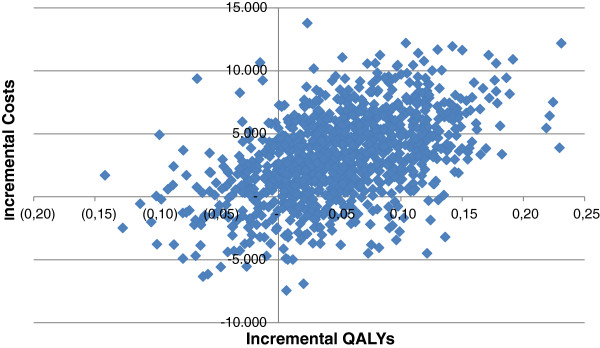
Cost-effectiveness plane of incremental costs and incremental effects for QALYs.

**Figure 4:**
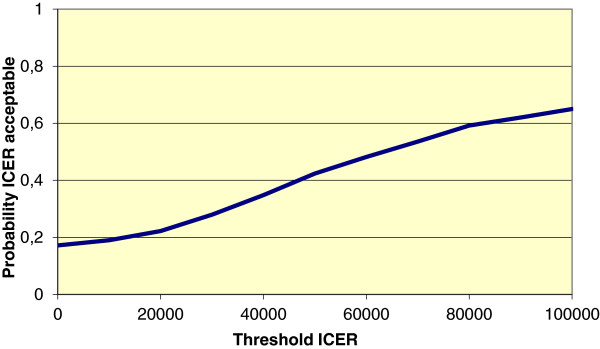
Cost-effectiveness acceptability curve for QALYs, ICER-approach.

Figure 3 shows the mean differences in costs and the mean difference in the outcome measure QALYs using 1000 bootstrap replicates of the trial data (differences based on IRR minus UC). This illustrates the uncertainty surrounding the estimates of expected cost-effectiveness (in €) between IRR and UC. In 10.6% of the simulations, IRR is both less effective and more costly, and hence unacceptable. Conversely, in 9.8% of the simulations IRR is both more effective and less costly and hence acceptable, regardless of societies willingness to pay (threshold) for a QALY. For all other simulated outcomes, the acceptability depends on the threshold cost-effectiveness ratio (ICER). Figure 4 shows for a range of threshold ICERs the proportion of simulated outcomes that is acceptable, or in other words, the probability that IRR is cost-effective compared to UC.

**Figure 5:**
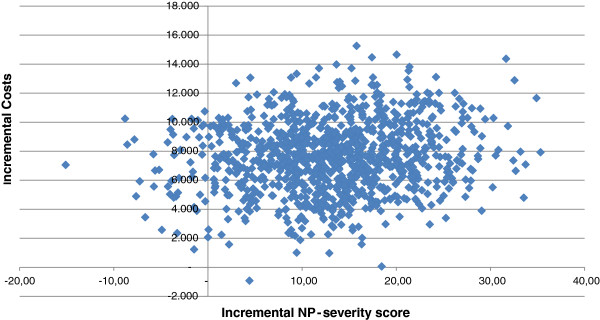
Cost-effectiveness plane of incremental costs and incremental effects for NPI-severity.

**Figure 6:**
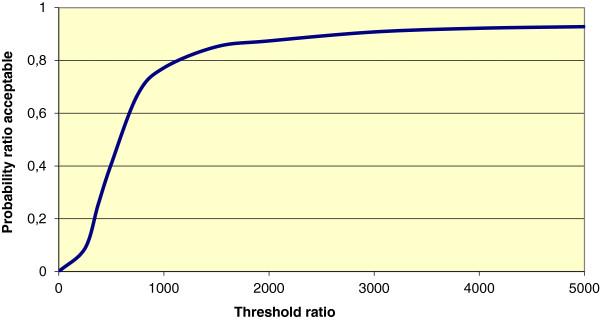
Cost-effectiveness acceptability curve for NPI-severity, ICER-approach.

Figure 5 shows the mean differences in costs and the mean difference in the outcome measure NPI-severity using 1000 bootstrap replicates of the trial data (differences based on IRR minus UC). This illustrates the uncertainty surrounding the estimates of expected cost-effectiveness (in €) between IRR and UC. In 4.2% of the simulations, IRR is both less effective and more costly, and hence unacceptable. Conversely, in 0.0% of the simulations IRR is both more effective and less costly and hence acceptable, regardless of societies willingness to pay (threshold) for improvement on NPI-severity. For all other simulated outcomes, the acceptability depends on a to be determined threshold cost-effectiveness ratio (ICER). Figure 6 shows for a range of threshold ICERs the proportion of simulated NPI-severity outcomes that is acceptable, or in other words, the probability that IRR is cost-effective compared to UC.

## Discussion

The objective of this study was an economic evaluation of an Integrative Reactivation and Rehabilitation (IRR-) programme. IRR was focused on psychogeriatric patients who were at high risk for admission to a nursing home. We estimated the cost-utility (QALYs) and cost-effectiveness (ICERs) of IRR by comparing IRR to usual multidisciplinary nursing home care (UC). The cost-utility analysis (CUA) shows that the amount of QALYs for psychogeriatric patients was almost equal between IRR and UC, while the costs were 10.5% lower in UC. Overall, the costs per QALY for IRR were far higher than € 30,000 per QALY (€ 275,000 per QALY) which the Dutch National Council for public health and health care assumes to be the threshold for cost-effectiveness of sustainable and meaningful care (2006) [38]. To test the sensitivity of the results for missing data we also applied the multiple imputation methodology, a technique commonly applied in economic evaluations [39,40]. Using multiple imputation, total medical costs in IRR were significantly higher than in UC (€ 4.572,--; 95% Cl: 364.24 to 8797.76) and the number of QALYs was non-significantly lower (0.02; 95% CI: -0.09 to 0.05). So, this analysis supported the conclusion that IRR apparently is not cost-effective in terms of QALYs. Moreover, CEAC of QALYs suggests that, when the costs per patient around € 60,000 is acceptable, then the probability is 0.50 that the treatment (i.c. IRR) is considered cost-effective compared to UC.

In contrast, at six-month follow-up, the cost-effectiveness analysis in terms of ICERs showed significant surplus improvement of patients (up to 34%) and caregivers (up to 50%) in IRR. Surplus improvement of the patient on severity of multiple psychiatric symptoms was about € 320 per point. For the caregivers, the surplus improvement on general burden was about € 130 per point and on competence € 540. These are clinically relevant results because MPS of patients and caregiver burden are the two phenomena experienced as most problematic in dementia care [41]. CEAC of MPS suggests that, when the costs per patient around € 600 is acceptable, then the probability is 0.50 that the treatment (i.c. IRR) is considered cost-effective compared to UC.

However, the cost-effectiveness results are not easily compared to cost-effectiveness of other health care interventions, because there is no reference value of costs per effect unit for these clinical outcomes. Hence, this type of information is of less value in health care policy decision making than costs per QALY.

The difference in results between QALYs and ICERs is remarkable. In terms of mean differences, the cost-effectiveness on clinically relevant outcomes, i.e. severity of psychiatric symptoms in psychogeriatric patients, caregiver burden and competence was relatively large and in favour of IRR [42,43]. In contrast, the mean difference between the IRR and UC on the EQ5D was minor (0.04; 95% CI: -0.06 to 0.14), with only relatively small percentages of clinically relevant (≥ 0.5 SD) improved patients (IRR 24% vs. UC 15%) [12,13]. This means that the EQ5D proved to be relatively unresponsive to change. This corresponds to the findings of Ballard et al. [44], who showed that clinically relevant improvement on Behavourial and Psychological Symptoms of Dementia (BPSD) had only small effects on general quality of life measurements. Completion of the EQ5D by proxy (i.e. the caregiver) may enhance the correlation between EQ5D and BPSD [35]. All in all, this does not facilitate comparison of the effects of interventions in cost-utility studies in psychogeriatrics. Further research is urgently needed [45-48].

One of the strengths of this study was that it was based on a relatively large sample size of patients. Furthermore, it was possible to estimate the benefit of full participation as the relatively high percentage of dropouts did not differ significantly on the observed variables. The majority of dropouts could be ascribed to death, which was not significantly different between IRR and UC. The phenomenon of high dropout percentages is well known in geriatric research [5-7]. It basically reflects the vulnerability of the psychogeriatric patients suffering from MPS.

How can the results be interpreted within the context of the literature? They confirm that psychotherapeutic treatment, based on person-oriented and problem-solving principles, is effective in psychogeriatric patients [46,48]. Moreover, the relative unresponsiveness of the patient’s EQ5D compared to the positive changes on the severity of MPS is in line with the results reported in literature [44-48]. Furthermore, as far as we know, our RCT-study was one of the first comprehensive studies in a nursing home setting with a relative large sample size to address integrative psychotherapeutic treatment of psychogeriatric patients and their caregivers [7,9].

However, the study had some weaknesses. First of all, RCT was not blinded. In a clinical study like this, blinding is not feasible. As the research staff had to visit the patients and caregivers personally, they knew the intervention history of the patients. Although we trained the research staff to administer the assessment instruments, this was no guarantee that information bias was precluded. Whether bias emerged in favour of IRR is difficult to demonstrate. As the assessments at baseline showed only minor differences between IRR and UC, except for somatic co-morbidity, the information bias at baseline seems to be limited. Another weakness was that only direct medical costs of patients were available; any other costs, specifically costs related to informal care at home, had to be excluded. This may have led to an underestimation of costs at home, especially in UC. A third limitation was the absence of (medical) cost-data for the caregiver. Especially, the significantly large beneficial effects of IRR on carer burden and competence may have lowered their medical consumption. This means that the results of the economic evaluation suggest a probable underestimation of (cost-)effectiveness of IRR. Furthermore, the study ended at 40 weeks.

Regarding generalization of the findings of this RCT, it is important to keep in mind that 50% of the eligible patients refused to participate. A relatively large proportion of these patients lived with a spouse. However, post-hoc prognostic analysis showed that living together did not have any prognostic quality with respect to improvement on the primary outcome variable. In IRR the beneficial long-term effects on the patient and the caregiver confirm those mentioned in the literature [46,48]. It is expected that by identifying the less effective therapeutic components and subsequently making them more effective or leaving them out, the cost-effectiveness of IRR may increase. Identification of and screening for those psychogeriatric patients and their caregivers with a relatively high likelihood of improving, presents another opportunity to increase the cost-effectiveness of IRR.

## Conclusion

In terms of QALYs, IRR did not seem to be cost-effective as compared to UC. However, at six-month follow-up, fully participating patients and caregivers in IRR improved significantly more on the clinically relevant parameters, i.e. severity of psychiatric symptoms (up to 34%) as well as on caregiver burden (up to 50%). Furthermore, the surplus effect on the competence of caregivers was substantial. The incremental costs-effectiveness ratios varied from € 130 to € 540 per additional point of improvement, on the multiple psychiatric symptoms (MPS) of the patient and caregiver burden and competence respectively. The total medical costs were 10.5% higher in IRR. The large discrepancies between IRR and UC on QALYs and ICERs demand further research regarding EQ5D validation in intervention studies with psychogeriatric patients. Considering all available evidence, the surplus costs of IRR may be considered acceptable when taking into account the beneficial effects on the high societal costs of suffering from multiple psychiatric symptoms of psychogeriatric patients and high burden of caregivers. To optimize the cost-utility and cost-effectiveness of IRR, the development of a tool to identify suitable psychogeriatric patients and caregivers for IRR is of key clinical and economic importance. Such a tool would contribute to the optimization of medical decision making based on an economical evaluation. Future studies that include caregiver costs have to be performed to strengthen the evidence, preferably RCTs.

## Competing interests

All authors declare that they have no financial of non-financial competing interests, nor are there intellectual or ideological controversies involved. The authors are independent researchers, affiliated with university-research departments, working together, and do not hold any shares in an organization that may gain of lose financially from the publication of this manuscript.

The authors are not affiliated in any way with/ did not receive any support from industries with commercial interests.

For this study a grant was received from The Netherlands Organization for Health Research and Development (http://www.zonmw.nl), a national organization that promotes quality and innovation in the field of health research and health care, initiating and fostering new developments. The majority of ZonMw’s commissions come from the Dutch Ministry of Health, Welfare and Sport (VWS).

## Authors’ contributions

LH-R was responsible for the statistical design of the study, and for carrying out the statistical analysis and wrote the article. TJEMB designed the study, was responsible for the statistical design of the study, carried out the research, supervised the data collection and wrote the article. MA was responsible for carrying out the statistical analysis. JL carried out the research, supervised the data collection, collected and analyzed the data. HJD designed the study, was responsible for the statistical design of the study, and for carrying out the statistical analysis, analyzed the data and wrote the article. MWR advised the researcher and reviewed the article. RH advised the researcher and reviewed the article. All authors read and approved the final manuscript.

## Pre-publication history

The pre-publication history for this paper can be accessed here:

http://www.biomedcentral.com/1472-6963/13/370/prepub
